# Lipotoxicity, Nutrient-Sensing Signals, and Autophagy in Diabetic Nephropathy

**DOI:** 10.31662/jmaj.2020-0005

**Published:** 2020-04-07

**Authors:** Shinji Kume, Hiroshi Maegawa

**Affiliations:** 1 Department of Medicine, Shiga University of Medical Science, Otsu, Japan

**Keywords:** Free fatty acid, proximal tubular cell, podocyte, lipotoxicity, proteinuria, autophagy

## Abstract

Diabetic nephropathy is a leading cause of proteinuria, kidney fibrosis, and subsequent end-stage renal disease. The renal prognosis of diabetic patients with refractory proteinuria is extremely poor. Therefore, identification of novel therapeutic targets to combat this serious condition and improve renal prognosis is urgently necessary. In diabetic patients, in addition to blood glucose levels, serum levels of free fatty acids (FFAs) are chronically elevated, even during postprandial periods. Of the various types of FFAs, saturated FFAs are highly cytotoxic and their levels are elevated in the serum of patients with diabetes. Thus, an increase in saturated FFAs is currently thought to contribute to proximal tubular cell damage and podocyte injury in diabetic nephropathy. Therefore, protecting both types of kidney cells from saturated FFA-related lipotoxicity may become a novel therapeutic approach for diabetic patients with refractory proteinuria. Interestingly, accumulating evidence suggests that controlling intracellular nutrient signals and autophagy can ameliorate the FFA-related kidney damage. Here, we review the evidence indicating possible mechanisms underlying cell injury caused by saturated FFAs and cell protective roles of intracellular nutrient signals and autophagy in diabetic nephropathy.

## 1. Introduction

Diabetic nephropathy is a world’s leading cause of end-stage renal disease, and its prevalence is increasing. Thus, to improve healthy lifespan in patients with diabetic nephropathy, a complete understanding of its pathogenesis and effective therapeutic strategies are required. Glomerulosclerosis and albuminuria are characteristic histological and clinical features of diabetic nephropathy. Recent clinical studies have demonstrated that intensive care treatment including strict glycemic controls and use of a renin-angiotensin system (RAS) antagonist can abolish or diminish low-grade proteinuria, such as microalbuminuria, in early-stage diabetic nephropathy^(1), (2), (3), (4)^. However, once proteinuria develops to massive and refractory proteinuria, the resultant tubulointerstitial damage leads to nephron loss and subsequent renal dysfunction. To better improve renal outcome in diabetic nephropathy, it is therefore necessary to identify new therapeutic targets to prevent stage progression from low-grade to massive and refractory proteinuria or to protect proximal tubular cells from proteinuria-related toxicity.

In addition to the above-mentioned current standard of approach to diabetic nephropathy, some emerging therapeutic agents have been becoming to be applied in clinical settings. Several anti-diabetic agents currently used in clinical settings may have some pleiotropic renoprotective effects. For instance, thiazolidinediones have direct renoprotective action experimental models ^(5)^, and their anti-albuminuric effect was observed in a meta-analysis ^(6)^. Inhibitors of dipeptidyl peptidase-4 (DPP-4) have emerged in the treatment paradigm of diabetes. Some experimental models have indicated possible renoprotective benefits of DPP-4 inhibitors ^(7)^, and actually, a clinical trial suggested anti-albuminuric effect of this type agent in type 2 diabetic patients treated with RAS inhibitors ^(8)^. Furthermore, a recent clinical trial using a sodium-glucose co-transporter 2 (SGLT2) inhibitor, empagliflozin, appears to improve the composite hard renal endpoints in type 2 diabetic patients at high cardiovascular risk ^(9)^, in which some pleiotropic effects such as persistent hyperketonemia ketone bodies and/or hematocrit are suggested to be involved in the renoprotective mechanism of the SGLT2 inhibitor ^(10)^. In addition, other drugs, which have been invested to ameliorate directly pathogenic responses such as inflammation, oxidative stress, and vasoconstriction, have recently been developed and expected as novel drugs for diabetic nephropathy. These drugs include selective C-C chemokine receptor type 2 antagonist ^(11)^, vitamin D receptor activators ^(12)^, nuclear respiratory factor 2 activator, bardoxolone methyl ^(13)^, and selective endothelin-A antagonism ^(14)^. Thus, aside from the current standard therapy, some other therapeutic options have been expected to improve renal outcome in diabetic nephropathy in the future. However, until their long-term safety and efficacy are ensured, their therapeutic effectiveness is still uncertain. Thus, we still need to identify a novel therapeutic target to improve renal outcome in diabetic nephropathy.

Insulin has a variety of biological effects. One of its important physiological roles is to facilitate glucose uptake into peripheral tissues and prevent the release of free fatty acids (FFAs) from adipose tissue during postprandial periods ^(15)^. In diabetes, reduced insulin action on skeletal muscle, the liver, and adipose tissue causes hyperglycemia and impairs the normally rapid postprandial decline of serum FFA levels. Thus, in addition to hyperglycemia, high FFA levels caused by insufficient insulin action represent a potential pathogenic factor in diabetic complications. Indeed, increasing experimental evidence supports a pathogenic role for FFAs in the progression of β cell dysfunction-related diabetic complications in late-stage diabetes ^(16), (17)^. Furthermore, experimental evidence has accumulated demonstrating a pathogenic role of FFAs, especially saturated FFAs, in podocyte damage, leading to massive proteinuria and proteinuria-related tubular cell damage in diabetic nephropathy. Thus, reducing saturated FFA-mediated cell toxicity may serve as an emerging therapy for refractory diabetic nephropathy. However, no drug and strategy aimed at ameliorating saturated FFA-mediated cell toxicity have not been developed in clinical settings.

In this review, we describe our reasons for focusing on FFA levels as a pathogenic factor in diabetic nephropathy and also discuss mechanisms underlying cell injury by saturated FFAs in this serious diabetic complication, which may help contribute to future development of novel drugs for diabetic nephropathy.

## 2. Altered FFA Metabolism in Diabetes

**Figure 1.**
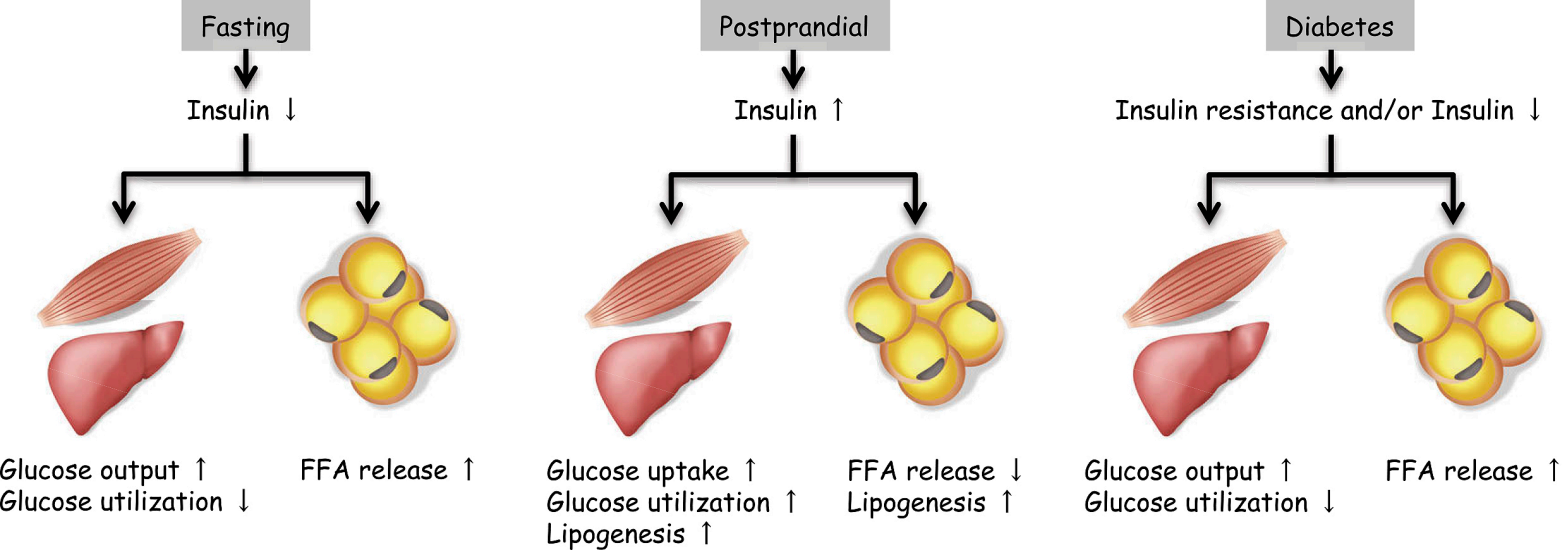
Glucose and free fatty acid (FFA) metabolism during fasting, the postprandial period, and diabetes. Insulin’s physiological action in glucose and lipid metabolism includes enhancing glucose uptake, glycogen synthesis and lipogenesis in peripheral tissues such as skeletal muscle and the liver and stopping gluconeogenesis in the liver and FFA release from adipose tissue. Thus, during fasting, decreased insulin secretion stimulates glucose production via gluconeogenesis in the liver and FFA release from adipose tissue and inhibits glucose utilization by skeletal muscle (left). In contrast, postprandial increases in insulin secretion stimulate glucose uptake, glucose utilization, and lipogenesis and inhibit FFA release (middle). In diabetes, insufficient insulin action due to insulin resistance and/or deficient insulin secretion causes hyperglycemia and sustained FFA release even during postprandial periods. Thus, hyperglycemia accompanied by sustained high levels of plasma FFA is a unique metabolic alteration only shown in diabetes (right). Hyperglycemia due to loss of insulin action to the liver and skeletal muscle has long been focused as a pathogenesis of diabetic vascular complications. Higher plasma FFA due to loss of insulin action to adipose tissue is another specific metabolic alteration in diabetes, which has been recently focused as a pathogenic factor in diabetic complications including nephropathy.

Insulin action on peripheral metabolic tissues decreases as a result of deficient insulin secretion in type 1 diabetes or insulin resistance combined with insufficient insulin secretion in type 2 diabetes. The physiological role of insulin in skeletal muscle and the liver is to facilitate glucose uptake and utilization during the postprandial period (Figure 1). Thus, impaired insulin action in these tissues causes the hyperglycemia that occurs in diabetes. Another important physiological action of insulin is its enhancement of lipogenesis and inhibition of FFA released by adipose tissue (Figure 1). Because FFAs are used as an energy source in various tissues during fasting, fasting serum FFA levels are very high in both diabetic and non-diabetic subjects. In non-diabetic subjects, serum FFA levels rapidly decrease during the postprandial period in an insulin-dependent manner. In contrast, in diabetic patients, who have impaired insulin action in adipose tissue, high FFA levels are sustained even during the postprandial period (Figure 1). Thus, in addition to hyperglycemia, high postprandial FFA levels may represent a typical metabolic alteration in diabetes. The risk of hyperglycemia in diabetic nephropathy has been well documented; it is now necessary to focus more on high FFA levels, also caused by insufficient insulin action, as a risk factor for diabetic nephropathy.

## 3. The Fraction of FFAs in Diabetic Complications

FFAs are classified into saturated, monounsaturated, and polyunsaturated fatty acids. These FFA classes have specific roles in metabolism and in the pathogenesis of diabetes-related diseases. Previous reports have demonstrated that diabetic patients exhibit abnormal fractions of serum FFAs, with proportionately higher levels of saturated FFAs compared with other types ^(18), (19)^.

A number of previous studies showed that saturated FFAs, such as palmitate and steric acid, induced cell damage and dysfunction in various tissues and that such damage was ameliorated by unsaturated FFAs, such as oleate (a monounsaturated fatty acid) and ω-3 polyunsaturated fatty acid ^(20), (21)^. These observations suggest involvement of an imbalance between FFA classes in the pathogenesis of diabetic complications and indicate the possibility that control of FFA type, as well as quantity, might represent a novel therapeutic approach for diabetic nephropathy.

## 4. Saturated FFAs in Diabetes-Related Proximal Tubular Cell Damage

Proteinuria is a major symptom of diabetic nephropathy and indicates impairment of the glomerular filtration barrier. Albumin, a major excreted macromolecule in proteinuria, is a carrier protein that binds to a number of molecules in the blood. Because serum FFAs bind to albumin, they are filtered through the impaired glomerular filtration barrier and reabsorbed by proximal tubular cells, along with albumin, in patients with refractory proteinuria. These albumin-bound FFAs then induce oxidative stress and inflammatory cytokine production in the proximal tubular cells, leading to tubulointerstitial damage and subsequent renal dysfunction ^(22), (23), (24), (25)^. Thus, protecting the proximal tubular cells from FFA-induced lipotoxicity is a potential therapeutic approach for improving the prognosis for renal function in diabetic patients with refractory proteinuria.

A pathogenic role of FFAs bound to albumin in the development of proximal tubular cell damage has been demonstrated by experimental studies in humans and animals. One such study showed that severe tubulointerstitial damage was induced by FFA-bound albumin, but not by FFA-free albumin, in a mouse experimental model ^(26)^. Furthermore, a human cohort study showed that diabetic patients with proteinuria in the nephrotic range had higher urinary levels of FFAs and more severe tubulointerstitial injury than patients with non-diabetic minimal-change nephrotic syndrome ^(27)^. A more recent paper describing a human cohort study using RNA microarrays demonstrated that lipid metabolism was altered in proximal tubular cells of patients with kidney diseases, in a manner strongly associated with the progression of their tubulointerstitial lesions ^(28)^. Therefore, chronic elevation of FFA levels is implicated in the proximal tubular cell damage caused by proteinuria in diabetic nephropathy.

## 5. Molecular Mechanisms Underlying Saturated FFA-Mediated Proximal Tubular Injury

**Figure 2.**
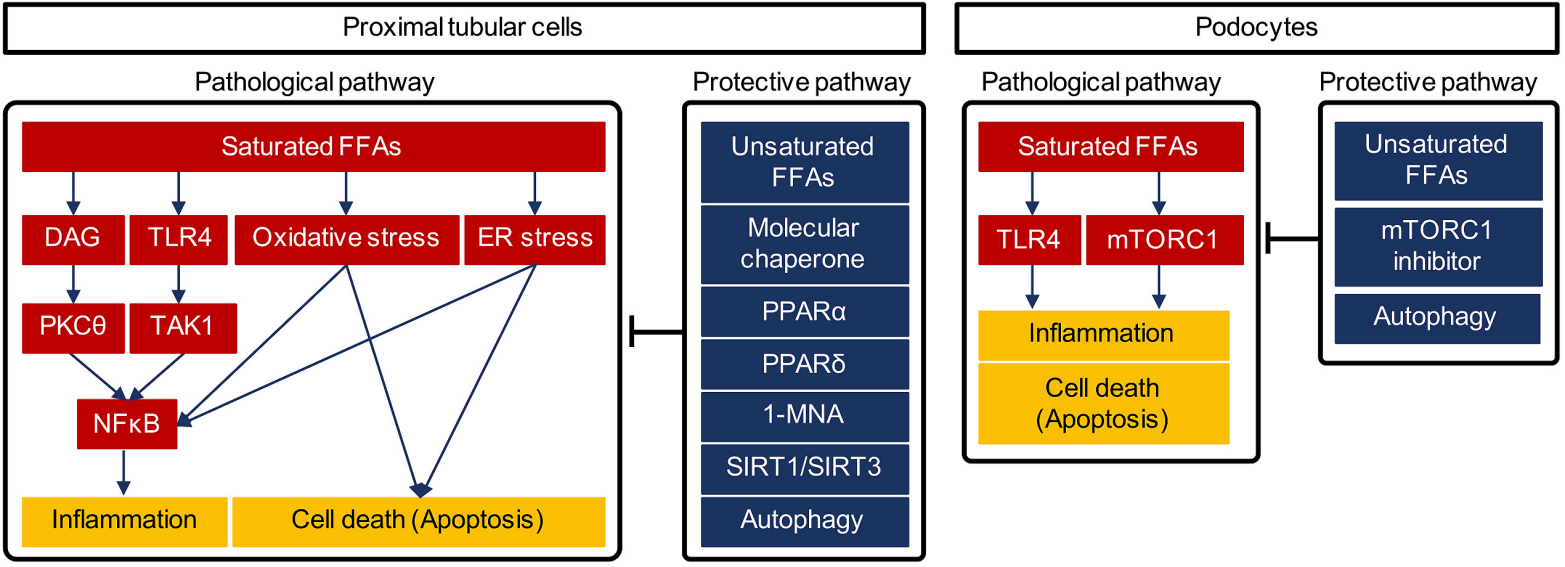
Proposal mechanism underlying saturated free fatty acids-induced cell damage. Saturated FFAs damage proximal tubular cells via multiple mechanisms such as DAG-PKCθ pathway, TLR4-dependent pathway, oxidative stress, and ER stress (left). In addition to unsaturated FFAs, several agonists or other methods to activate certain molecules and cellular system including autophagy can antagonize cell damage by saturated FFAs. Similarly, saturated FFAs damage podocytes via activation of TLR4 and mTORC1 signal (right). Unsaturated FFAs, mTORC1 inhibitor, and autophagy activation may be useful to prevent the damage. FFAs, free fatty acids; DAG, diacylglycerol; PKC, protein kinase C; TLR4, toll-like receptor 4; 1-MNA, 1-methylnicotinamide. SIRT; silent information regulator; ER, endoplasmic reticulum; PPAR, peroxisome proliferator-activated receptor.

We propose several molecular mechanisms that could underlie saturated FFA-mediated proximal tubular injury (Figure 2).

Diacylglycerol (DAG) is a well-known pathogenic factor in diabetic complications. Hyperglycemia increases DAG formation, leading to hyper-activation of protein kinase C isoforms (PKCs) ^(29)^. Activation of PKC signaling is strongly associated with fibrosis, cell death, and inflammation, and its inhibition is considered a potential therapeutic approach to diabetic nephropathy ^(30)^. Our recent study showed that palmitate, a saturated FFA, led to increased intracellular DAG accumulation and subsequent activation of PKCθ in proximal tubular cells ^(20)^. Saturated FFA-mediated activation of the DAG-PKCθ pathway induced an inflammatory response via upregulation of NFκB and mitochondrial cell death in cultured proximal tubular cells ^(20)^. Thus, high levels of saturated FFAs may also contribute to activation of DAG-PKC pathways in human diabetic nephropathy, even though such activation has long been regarded as being mediated primarily by hyperglycemia.

Toll-like receptor 4 (TLR4) is implicated in the recognition of various bacterial wall components ^(31)^. Activation of toll-like receptor-mediated signaling can induce inflammation. Recent reports showed that saturated FFAs can act as endogenous ligands for TLR4, which, when activated, induces inflammation and contributes to development of several diseases ^(32), (33)^. Proximal tubular cells express TLR4 along the brush border ^(34)^. We previously reported that palmitate activated TLR4 signaling and induced inflammation via activation of the signaling pathway downstream of TLR4 in proximal tubular cells ^(35)^. Interestingly, peroxisome proliferator-activated receptor (PPAR) δ agonists exerted anti-inflammatory effects via inactivation of transforming growth factor β-activated kinase (TAK1), downstream of TLR4. TAK1 inactivation inhibited NFκB activation by enhancing degradation of IκB, an NFκB suppressor ^(35)^. A PPAR δ agonist, GW501516, inhibited NFκB-mediated overexpression of MCP-1 by directly inhibiting TAK1 activity ^(35)^. Thus, although PPARδ agonists were originally developed as activators of mitochondria function and fatty acid oxidation, they are likely to have a pleiotropic effect as a direct inhibitor of TLR4-mediated inflammation. PPARδ agonists may therefore represent novel potential therapeutic agents for protection of proximal tubular cells from FFA-related lipotoxicity.

Fenofibrate is a PPARα agonist used clinically for treating hypertriglycemia. Experimental evidence demonstrated that this agent ameliorated proximal tubular injury induced by palmiate ^(23)^ and that PPARα deficiency in mice exacerbated kidney injury induced by FFA-bound albuminuria ^(36)^. In contrast to the renoprotective mechanism of PPARδ agonists, PPARα inhibited palmitate-induced proximal tubular cell injury by enhancing fatty acid oxidation and subsequently reducing saturated FFA-related lipotoxicity in both mice and cultured proximal tubular cells ^(23)^. A clinical study, the Fenofibrate Intervention and Event Lowering in Diabetes study, showed that the PPARα agonist fenofibrate prevented initiation and progression of albuminuria in patients with type 2 diabetes ^(37), (38)^. Unfortunately, fenofibrate cannot be given to patients with renal dysfunction because it is renally excreted. However, for patients with normal renal function, PPARα agonists such as fenofibrate might be useful for treating early diabetic nephropathy.

Endoplasmic reticulum (ER) stress is also recognized as a pathogenic factor in diabetic complications ^(39)^. One study found that palmitate stimulation induced excessive ER stress and activation of C/EBP homologous protein, leading to cell death, in cultured proximal tubular cells ^(40)^. Supplementation with molecular chaperones to increase protein folding capacity in the ER reduced this palmitate-induced proximal tubular cell damage ^(40)^. These findings indicate that enhancing ER capacity by molecular chaperone supplementation might be a therapeutic approach for diabetic nephropathy.

Nicotinamide adenine dinucleotide (NAD) metabolism has recently been considered as a potential therapeutic target for several diseases, including diabetes and age-related neurodegenerative disorders ^(41), (42), (43)^. Our recent work showed that palmitate increased expression of nicotinamide n-methyltransferase, an NAD metabolizing enzyme ^(44)^. The product of nicotinamide n-methyltransferase activity, 1-methylnicotinamide, reduced mitochondrial oxidative stress and cell death in FFA-induced renal tubular damage ^(44)^. 1-methylnicotinamide may, therefore, serve as a treatment for lipotoxicity-mediated renal injury in diabetic patients with refractory proteinuria.

Calorie restriction extends lifespan in all organisms and is renoprotective. It also has anti-obesity and anti-diabetic effects in mammals ^(45)^. Studies designed to identify factors responsible for life extension by calorie restriction have identified a number of potential anti-aging molecules. Of these, sirtuins, which are known to induce autophagy, are now the focus of approaches to prolonging healthy lifespan ^(46), (47)^. Interestingly, several recent studies, including ours, showed that sirtuin activity and the process of autophagy are both crucial for renoprotection in diabetic nephropathy.

Sirtuins are NAD-dependent deacetylases and are classified into seven types in mammals ^(46)^. These enzymes sense changes in intracellular NAD concentrations and control cellular activity in response to intracellular energy state. Among these enzymes, SIRT1 has been suggested as a potential therapeutic target in several conditions, such as diabetes and kidney disease ^(48), (49), (50)^. Indeed, one study showed that SIRT1 overexpression, specifically in proximal tubular cells, ameliorated progression of diabetic nephropathy in mice ^(51)^. Furthermore, some SIRT1 activators, including resveratrol, have been shown to inhibit development of diabetic nephropathy in mice ^(52)^. Thus, SIRT1 activation is currently under investigation as a therapeutic approach for various diseases, including diabetes and its vascular complications.

In addition to SIRT1, SIRT3, which is localized in mitochondria, is associated with alleviating lipotoxicity-mediated tubular injury. Interestingly, SIRT3 expression levels have been shown to decrease in mouse kidneys damaged by FFA-bound proteinuria and in cultured proximal tubular cells treated with palmitate ^(22)^. Furthermore, decreased SIRT3 expression level was associated with increased MCP-1 expression in the kidneys damaged by FFA-bound proteinuria, whereas overexpression of SIRT3 reduced reactive oxygen species accumulation by enhancing mitochondrial oxidative capacity in proximal tubular cells exposed to palmitate ^(22)^. Thus, decreased SIRT3 expression may be involved in the pathogenesis of lipotoxicity-mediated tubular cell damage, and activation of SIRT3 may be another potential therapeutic target, although no specific SIRT3 activator is currently available.

Autophagy is an intracellular degradation system that maintains intracellular homeostasis by removing damaged proteins and organelles during stress conditions, including starvation, hypoxia, and ER stress ^(53)^. Autophagy machinery is needed across species to overcome starvation ^(54)^, and its alteration is involved in the pathogenesis of many diseases including diabetes and neurodegenerative diseases ^(53)^. Interestingly, there is increasing evidence that autophagy exerts a renoprotective effect during various forms of renal toxic stress, aging, ischemia, and treatment with anti-cancer drugs ^(55)^. We recently reported that autophagy activity was suppressed in the kidneys of mice with high-fat diet (HFD)-induced obesity and type 2 diabetes, although the detailed factor to inhibit the activity in the kidney ^(56)^. More importantly, suppression of autophagy exacerbated proteinuria-related tubular cell damage in obese, type 2 diabetic, proximal tubular cell-specific autophagy-deficient mice ^(56)^. HFD feeding is a standard model for insulin resistance ^(56)^. Therefore, autophagy insufficiency under conditions of insulin resistance, such as in type 2 diabetes, may be a crucial molecular mechanism underlying lipotoxicity-mediated proximal tubular cell injury. Furthermore, autophagy activators may therefore be possible treatments for diabetic nephropathy.

## 6. Saturated FFAs in Diabetes-Related Podocyte Damage

Proteinuria is caused by disruption to the glomerular filtration barrier that is maintained by the podocyte. In recent reports, podocytes were shown to be damaged by saturated FFAs, via similar molecular mechanisms as proximal tubular cells, in diabetic nephropathy. Thus, saturated FFA-related lipotoxicity is likely to be associated with initial proteinuria pathogenesis, as well as with the tubulointerstitial lesions associated with proteinuria in diabetic nephropathy.

As mentioned previously, saturated FFAs have been shown to activate TLR4 and induce inflammation in proximal tubular cells. In cultured podocytes, high glucose with saturated FFAs has been shown to increase TLR4 expression and proinflammatory cytokine synthesis ^(57)^. Inhibition of the TLR4 signaling pathway has been demonstrated to abolish saturated FFA-induced proinflammatory cytokine synthesis, which indicates a potential renoprotective role for TLR4 inhibition in diabetic podocytopathy. C/EBP homologous protein is a marker of activated ER stress and induces ER stress-related apoptosis. Expression of this protein and subsequent apoptosis were increased in cultured podocytes treated with saturated FFAs, and these effects were attenuated by co-treatment with unsaturated fatty acids ^(58)^. These pathogenic mechanisms are very similar to those demonstrated in the proximal tubular cell study.

In addition, we recently proposed a renoprotective mechanism associated with calorie restriction in another podocyte study. The mammalian target of rapamycin (mTOR) complex 1 (mTORC1) is a nutrient-sensing kinase that regulates a wide range of cellular processes and can sense hyper-nutrient states such as hyperglycemia and amino acid sufficiency ^(59)^. Although mTORC1 is physiologically essential for cell growth, differentiation, protein translation, and inhibition of autophagy, its hyper-activation was recently reported to be strongly associated with pathogenesis of diabetic podocytopathy ^(60), (61), (62)^. Thus, revealing the molecular mechanisms underlying diabetes-related mTORC1 activation may inform development of new therapies for diabetic nephropathy. Our recent study showed that palmitate activated mTORC1, leading to podocyte apoptosis and that this was antagonized by oleate, a monounsaturated FFA ^(21)^. Thus, imbalances between FFA classes in diabetic patients may cause diabetes-related hyper-activation of mTORC1 in podocytes and subsequent cell damage.

Moreover, podocyte-specific autophagy-deficient mice developed podocyte loss and massive proteinuria in an HFD-induced diabetic model for inducing minimal proteinuria ^(63)^. Because mTORC1 is a potent inhibitor of autophagy, autophagy insufficiency may be involved in the mTORC1-dependent podocytopathy that occurs in diabetes. Thus, similar to the results in proximal tubular cells, altered intracellular nutrient-sensing signals and autophagy are likely to be involved in diabetic podocytopathy ^(64), (65)^. These findings may contribute to the development of new therapeutic strategies for diabetic podocytopathy.

## 7. Conclusion

We have reviewed the mechanisms underlying FFA-mediated damage in proximal tubular cells and podocytes. This overall injury process may provide novel therapeutic targets to improve renal outcome in diabetic patients with refractory proteinuria. Although several molecular mechanisms of saturated FFA-mediated kidney injury have recently been revealed, there is currently no treatment available. Thus, further basic and clinical research in this field is needed. Our review should provide useful knowledge and perspectives for future studies on the relationship between FFA-related lipotoxicity and the pathogenesis of diabetic nephropathy.

## Article Information

### 

This article is based on the study, which received the Medical Research Encouragement Prize of The Japan Medical Association in 2019.

### Conflicts of Interest

None

### Sources of Funding

This work was supported by Grants-in-Aid for Scientific Research (KAKENHI) from the Japan Society for the Promotion of Science grant number 25713033 to S. K.

### Acknowledgement

The author thanks all members of the Department of Medicine, Shiga University of Medical Science, for their supports.
